# Beyond the Bench: Tox-in-a-Box

**Published:** 2005-03

**Authors:** Kris Freeman

The Tox Ambassador—a scientist visiting from a nearby university—squeezes several drops of blue dye into three different-sized beakers of water. The audience of sixth-grade students can easily see that although the same amount of dye is put in each beaker, the water in the smallest “baby” beaker turns much darker than that in the larger “big kid” and “adult” beakers. They are learning a key concept in toxicology: that your body size affects your dose, and that the dose makes the poison.

Thousands of students have learned about environmental health science through presentations like these based on the Tox-in-a-Box™ resources kit, which includes activities for grades kindergarten through 12 (K–12), slides, demonstrations, instructions, tips for presenters, and a resource manual for teachers. The kit is designed for toxicologists and other environmental and public health professionals—Tox Ambassadors—to use in outreach efforts to students and teachers. It is produced and distributed by the Community Outreach and Education Program (COEP) of the NIEHS Center for Ecogenetics and Environmental Health at the University of Washington.

Tox-in-a-Box was originally developed by COEP staff at the request of environmental health professionals who had been invited to give presentations in classrooms and weren’t sure how to explain toxicology to young audiences. Soon COEP staff also began training graduate students to serve as Tox Ambassadors. Since 2000, Tox Ambassadors have given presentations to almost 4,000 K–12 students in western Washington. Many of these presentations have been made through the NIEHS/University of Washington Integrated Environmental Health Middle School Project (http://depts.washington.edu/ceeh/Outreach/k12.html).

When Carlos Mata, a former visiting scholar and Tox Ambassador, returned to his native country of Costa Rica, he launched a program similar to Tox-in-a-Box called EcoAmigos*.* The EcoAmigos program disseminates basic environmental health information in regional schools. Tox-in-a-Box kits have also been sent to collaborators at the University of Alexandria in Egypt.

Tox-in-a-Box fits well with many modules such as environment, biology, chemistry, social studies, risk, safety, reading, and debate. Learning themes include Living in a Chemical World, Routes of Exposure, Risks vs. Benefits, and Tox Tales: A Real Life Component. Overall, teachers give the kit high marks for both the relevance of the material to the students’ interests and concerns, and the ease of integrating the material into the curriculum they already teach.

Tox-in-a-Box kits are available for purchase. A brochure is available online at http://depts.washington.edu/ceeh/Outreach/pdf/tib.pdf.

## Figures and Tables

**Figure:**
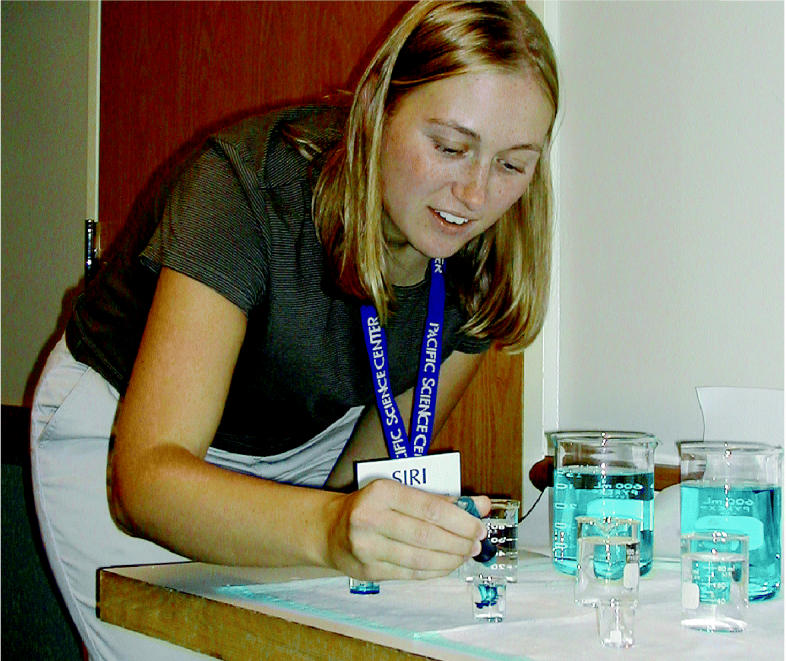

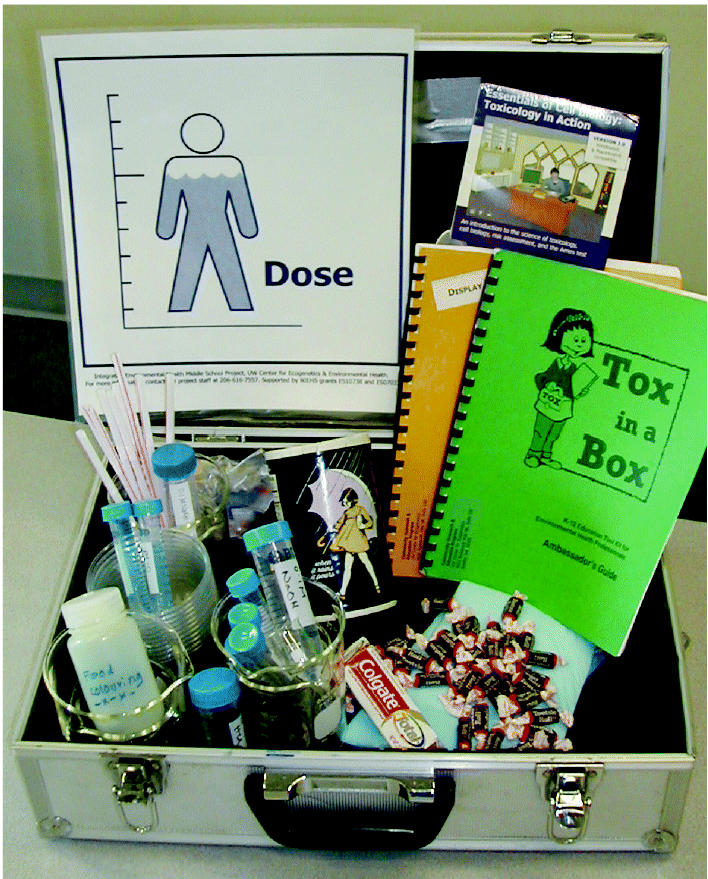
**Good things in small packages.** The Tox-in-a-Box kit (top) has everything needed for a school presentation (left).

